# Impact of the COVID-19 lockdown period on hospital admissions for paediatric accidents: a French nationwide study

**DOI:** 10.1007/s00431-024-05900-0

**Published:** 2024-12-04

**Authors:** Morgan Recher, Soxna F. K. Fall, Marguerite Lockhart-Bouron, Laure Lacan, Didier Theis, Stéphane Leteurtre, Amélie Bruandet

**Affiliations:** 1 Univ. Lille, CHU Lille, ULR 2694 – METRICS: Évaluation des technologies de santé et des pratiques médicales, 59000 Lille, France; 2 Department of Health Informatics, Lille University Medical Centre, Lille, France; 3 Réanimation et Soins Intensifs Pédiatriques Polyvalents, Hôpital Jeanne de Flandre, CHU de Lille, Avenue Eugène Avinée, 59037 Lille Cedex, France

**Keywords:** COVID-19, Paediatric, Accident, Paediatric intensive care

## Abstract

During the COVID-19 pandemic, lockdown posed a number of unprecedented problems. Accidents to children are a major public health concern. This study sought to determine the impact of France’s 2020 COVID-19 lockdown on hospital admissions for paediatric accidents. This study was a retrospective cohort analysis of data from the French national inpatient database. Children admitted to a French hospital for an accident from January to July 2020 and 2019 were included. The number of admissions for accidents between January and July in 2020 (the study period) vs. the same period (the control period) in 2019 was recorded. Hospital admission rate ratios (HRRs) comparing 2020 with 2019 were calculated for three periods in 2020 and 2019: before lockdown (P1, January 1 to March 15), during lockdown (P2, March 16 to May 10), and after lockdown (P3, May 11 to July 31). A total of 72,089 children were admitted to hospital for accidents between January and July 2020 (mean (SD) age, 8.7 (5.8)), and 84,961 between January and July 2019 (mean (SD) age, 9.2 (5.7)). A total of 12,864 children (18%) were admitted during the lockdown period (P2) in 2020, and 23,098 (27%) during the equivalent period in 2019 (HRR, 0.56; 95% confidence interval (CI), 0.55–0.57; *p* < .001). There were significantly fewer admissions for accidents in 2020 than in 2019; the HRR was 0.97 (95% CI, 0.96–0.99; *p* = .003) for P1 and 0.95 (95% CI, 0.93–0.96; *p* < .001) for P3. During the 2020 lockdown period (P2), the risk of accident-related paediatric admissions with critical care lasting for at least 1 day was lower than in 2019 (HRR 0.67; 95% CI, 0.62–0.73; *p* < .001).

*Conclusions*: Further research could usefully examine how parents create a safe home environment for their children.

**Table:** 

**What is Known:** • *During the COVID-19 pandemic, lockdown posed a number of unprecedented problems. Accidents to children are a major public health concern. This study sought to determine the impact of France’s 2020 COVID-19 lockdown on hospital admissions for paediatric accidents.*
**What is New:** • *The first nationwide COVID-19 lockdown in France was associated with a decrease in the absolute number of hospital admissions for paediatric accidents, for all age groups, as well as the number of accident-related paediatric hospital stays involving critical care.*

**Supplementary Information:**

The online version contains supplementary material available at 10.1007/s00431-024-05900-0.

## Introduction

The coronavirus disease 2019 (COVID-19) pandemic was an international public health emergency. Guided by the World Health Organization, many countries implemented periods of “lockdown” to control the spread of the disease. In France, the first strict lockdown period lasted from March 16 to May 10, 2020. Following the introduction of emergency legislation, people were confined to their homes, and outdoor movement was restricted to essential activities (including medical care).

Most accidents involving children occur (i) during domestic incidents (include poisoning, burns, suffocation by foreign bodies, falls, and drowning) at home or in the immediate surroundings (the garden, yard, or garage), (ii) away from home (e.g. at school or during sports activities), and (iii) on vacation [1]. The French Academy of Medicine has reported that, since the implementation of COVID-19 containment measures, the emergency services, firefighters, and media have reported an increase in domestic accidents [2]. However, the data on the number of children attending hospitals due to accident during the lockdown period are contradictory, with both increases and decreases reported [3, 4]. Moreover, all studies of this topic in France were conducted at single centres, and nationwide data are lacking [5, 6].

In view of these gaps in our knowledge, we decided to conduct a large, nationwide, population-based cohort study. We hypothesized that the number of accident-related hospital admissions among children and teenagers would decrease during France’s first period of nationwide COVID-19 lockdown due to changes in social behaviour and mobility. The primary objective was to describe the impact of France’s first period of COVID-19 lockdown on the number of accident-related hospital admissions of children. The secondary objectives were to perform stratified analyses by age group.

## Methods

### Data source

In the present retrospective cohort study, we analyzed data extracted from the French national inpatient database (*Programme de Medicalisation des Systèmes d’Information* (PMSI)) [7]. The PMSI is populated with information on diagnoses and medical procedures from standardized hospital discharge reports. The coding and reporting of this information are mandatory in all hospital departments. The discharge report abstracts in the PMSI include the patients’ demographic characteristics, diagnostic codes (based on the International Statistical Classification of Diseases and Related Health Problems, Tenth Revision [ICD-10]), and medical procedure codes (based on the French *Classification Commune des Actes Médicaux*).

To ensure that the data are of high quality, routine tests are performed when information on inpatient stays is sent to the French public health insurance agency. These tests include checks on the chronology of the hospital stays, the format of the demographic characteristics (missing, incorrect or inaccurate values for the sex, age, date, mode of entry, and date and mode of discharge), the format and accuracy of procedure and diagnostic codes, and the coherence of the relationships between procedure codes, diagnostic codes, the length of hospital stay, age, and sex.

The University of Lille institutional review board waived the need for informed consent because the study used de-identified medical data and did not involve human participant research. The study was reported in line with the Strengthening the Reporting of Observational Studies in Epidemiology guidelines.

### Participants

The study included paediatric patients (from 1 day to 17 years of age) admitted to hospital for accidents (including domestic accidents like trauma, intoxication, poisoning, and drowning) in metropolitan France from January 1 to July 31, 2019 (the control period), and from January 1 to July 31, 2020 (the study period). The diagnostic codes are given in eTable 1. The four age subgroups studied were below 1 year of age, 2 to 5, 6 to 12, and 13 to 17, as previously described in the literature [4, 6, 19]. Patients who had an accident but were not hospitalized were not recorded in this inpatient database.

The exclusion criteria were an incorrect French diagnosis-related group and the deaths of newborns within the first 24 h after birth. Consecutive hospital stays for which the time interval between stays was less than 1 day were considered to be a single stay. The start of the care sequence was defined as the starting date of the first stay, and the end date was defined as the date of death or the date of discharge at the end of the last stay.

### Exposures of interest

The main exposure of interest was the year 2020 vs. the year 2019. For the 2 years, three periods (Ps) were considered: January 1 to March 15 (before the lockdown, referred to hereafter as P1), March 16 to May 10 (during the lockdown, P2), and May 11 to July 31 (after the lockdown, P3). The patient’s sex, age, and hospital status were reported, together with whether the patient had died during the hospital stay. Critical care was defined as admission to a paediatric intensive care unit (PICU) or a paediatric intermediate care unit. During a given hospital stay, a patient could be admitted to both a PICU and a paediatric intermediate care unit.

The primary outcome was the number of accident-related hospital stays lasting 1 day or more for children in France. The secondary outcome was the number of these admissions involving critical care.

### Statistical analysis

Categorical variables were expressed as the frequency (percentage), and continuous variables were expressed as the mean (standard deviation (SD)) in the case of normal distribution and median (interquartile range) in the case of abnormal distribution. Normality of distribution was assessed using the Shapiro–Wilk test. χ2 tests were used to compare qualitative data, and non-parametric Mann–Whitney tests were used for quantitative data. The hospital admission rate ratio (HRR) for the study period vs. the control period was calculated for P1, P2, and P3, i.e. before lockdown, during lockdown, and after lockdown, respectively. The secondary outcomes were the changes in HRRs stratified by age group. For each interval and each modality of the secondary outcome variables, HRRs comparing the study period with the control period were calculated by using Poisson regression to model the overall number of hospital admissions, as previously described [8]. The accident rate for all patients and for each age group was calculated using the absolute number of patients relative to the total number of hospital admissions (eMethods). All tests were two-tailed, and the threshold for statistical significance was set to *p* < 0.05. All statistical analyses were performed using R software (version 4.3.0) [9].

## Results

### Characteristics of patients admitted for accidents from January to July in 2020 vs. 2019

In France, a total of 166,126 hospital stays for paediatric accidents were recorded from January 1 to July 31, 2019, and from January 1 to July 31, 2020 (Table 1).

**Table 1:** Characteristics of patients with paediatric accidents before, during, and after the first period of COVID-19 lockdown in France

	Total			Before lockdown (P1)			During lockdown (P2)			After lockdown (P3)		
Characteristics	2019	2020	*p* value	2019	2020	*p* value	2019	2020	*p* value	2019	2020	*p* value
All (*n*)	84,961	72,089		26,268	25,584		23,098	12,864		35,595	33,641	
Age (year)			< 0.0001			0.3230			< 0.0001			< 0.0001
Median (IQR)	10.0 (4.0–15.0)	9.0 (3.0–14.0)		11.0 (4.0–15.0)	11.0 (4.0–15.0)		10.0 (4.0–15.0)	6.0 (2.0–12.0)		10.0 (4.0–14.0)	9.0 (3.0–14.0)	
Range	0.0–17.0	0.0–17.0		0.0–17.0	0.0–17.0		0.0–17.0	0.0–17.0		0.0–17.0	0.0–17.0	
Age categories *n* (%)			< 0.0001			0.1818			< 0.0001			< 0.0001
Below 1 year	12,073 (14.2)	11,682 (16.2)		3894 (14.8)	3813 (14.9)		3151 (13.6)	2532 (19.7)		5028 (14.1)	5337 (15.9)	
2–5 years	14,950 (17.6)	13,852 (19.2)		4275 (16.3)	3991 (15.6)		4152 (18.0)	3351 (26.0)		6524 (18.3)	6509 (19.3)	
6–12 years	25,183 (29.6)	21,221 (29.4)		7092 (27.0)	6906 (27.0)		6895 (29.9)	3941 (30.6)		11,196 (31.5)	10,374 (30.8)	
13–17 years	32,754 (38.6)	25,335 (35.1)		11,007 (41.9)	10,874 (42.5)		8900 (38.5)	3040 (23.6)		12,847 (36.1)	11,421 (33.9)	
Sex *n* (%)			0.9866			0.1655			< 0.0001			0.3153
Girl	33,984 (40.0)	28,833 (40.0)		10,999 (41.9)	10,559 (41.3)		9285 (40.2)	5449 (42.4)		13,701 (38.5)	12,824 (38.1)	
Length of hospital stay			< 0.0001			< 0.0001			0.2957			0.0592
Median (IQR)	1.0 (0.0–1.0)	1.0 (0.0–1.0)		1.0 (0.0–2.0)	1.0 (0.0–1.0)		1.0 (0.0–1.0)	1.0 (0.0–1.0)		1.0 (0.0–1.0)	1.0 (0.0–1.0)	
Length of hospital stay (days in classes) (%)			< 0.0001			0.0001			0.0002			0.0068
< 1	32,781 (38.6)	28,306 (39.3)		10,199 (38.8)	10,405 (40.7)		8922 (38.6)	4924 (38.3)		13,660 (38.4)	12,977 (38.6)	
1 < 2	32,106 (37.8)	27,598 (38.3)		9381 (35.7)	8969 (35.1)		8722 (37.8)	5107 (39.7)		14,003 (39.3)	13,522 (40.2)	
2 < 3 days	8540 (10.1)	6998 (9.7)		2587 (9.8)	2448 (9.6)		2257 (9.8)	1219 (9.5)		3696 (10.4)	3331 (9.9)	
≥ 3	11,533 (13.6)	9188 (12.7)		4101 (15.6)	3762 (14.7)		3197 (13.8)	1614 (12.5)		4236 (11.9)	3811 (11.3)	
Death in hospital *n* (%)	163 (0.2)	120 (0.2)	0.2370	49 (0.2)	46 (0.2)	0.8576	39 (0.2)	28 (0.2)	0.3035	75 (0.2)	46 (0.1)	0.0199
Admissions with critical care *n* (%)	5770 (6.8)	5294 (7.3)	< 0.0001	1784 (6.8)	1740 (6.8)	0.9654	1552 (6.7)	1049 (8.2)	< 0.0001	2435 (6.8)	2504 (7.4)	0.0021
Admissions to a PICU *n* (%)	2005 (2.4)	1801 (2.5)	0.0757	637 (2.4)	583 (2.3)	0.2721	529 (2.3)	351 (2.7)	0.0099	839 (2.4)	867 (2.6)	0.0618
Admissions to a paediatric intermediate care unit *n* (%)	4141 (4.9)	3868 (5.4)	< 0.0001	1287 (4.9)	1271 (5.0)	0.7190	1118 (4.8)	772 (6.0)	< 0.0001	1737 (4.9)	1824 (5.4)	0.0012
Surgery *n* (%)	42,335 (49.8)	35,770 (49.6)	0.4051	12,473 (47.5)	12,401 (48.5)	0.0243	11,480 (49.7)	6433 (50.0)	0.5774	18,383 (51.6)	16,935 (50.3)	0.0006

*SD*, standard deviation; *IQR*, interquartile range; *PICU*, paediatric intensive care unit

**Fig. 1:**
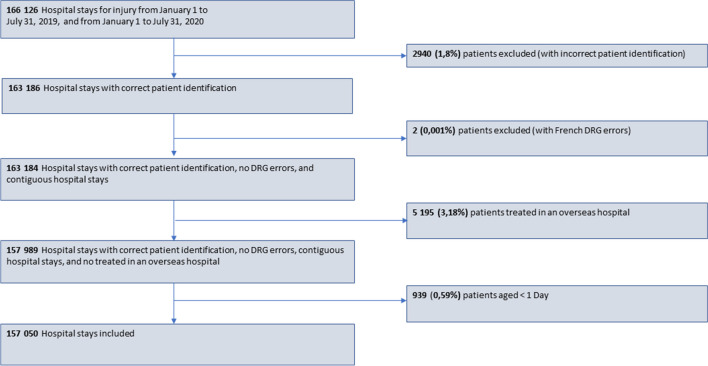
Study flow chart. Consecutive hospital stays for which the time interval between stays was less than 1 day were considered to be a single stay. DRG, diagnosis-related group

After the exclusion of stays that did not meet the study’s criteria, 157,050 hospital stays were included in our analysis (Fig. 1).

In 2020, 72,089 hospital stays for accidents were recorded; the median (IQR) length of stay was 1.0 (0.0–1.0) days). In 2019, 84,961 hospital stays for accidents were recorded; the median (IQR) length of stay was 1.0 (0.0–1.0) days).

**Fig. 2:**
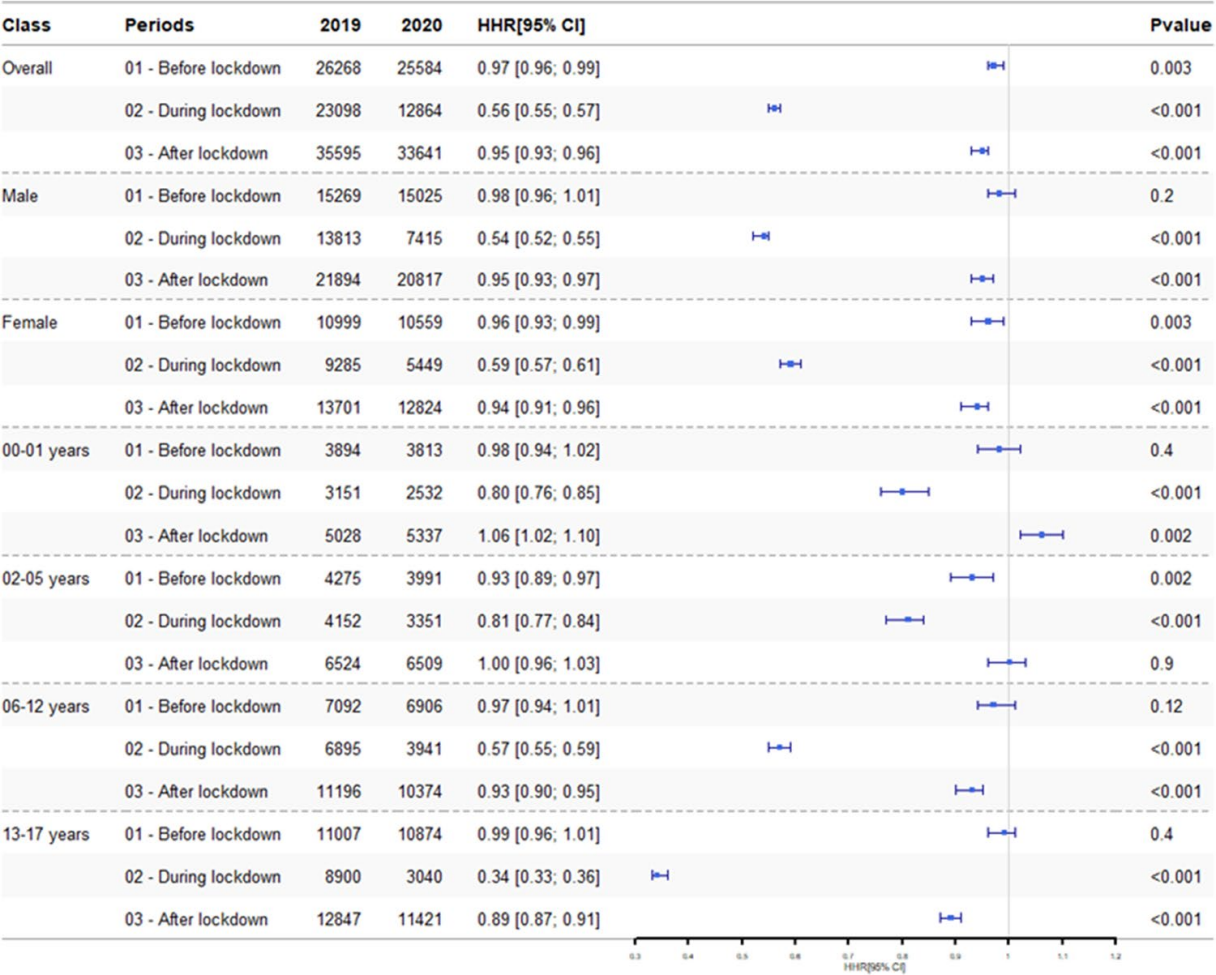
Hospital admission rate ratios (HRR) for paediatric accidents by age and by sex, from January 1 to July 31, 2019, and from January 1 to July 31, 2020

The number of hospitals stays for accidents was 12,864 for P2 in 2020 and 23,098 for P2 in 2019 (HRR, 0.56; 95% CI, 0.55–0.57; *p* < 0.001) (Fig. 2).

Relative to the total number of hospital admissions, the accident rates by diagnosis remained stable between 2019 and 2020 (eTable 2). During P2, the mortality rates were low and similar in 2020 and 2019 (0.2%), and the year-on-year difference in the incidence of surgery (50% in 2020 vs. 49.7% in 2019) was not significant.

For P1 and P3, there were significantly fewer admissions for accidents in 2020 than in 2019; the HRR was 0.97 (95% CI, 0.96–0.99; *p* = 0.003) for P1 and 0.95 (95% CI, 0.93–0.96; *p* < 0.001) for P3 (Fig. 2).

### Accident-related hospital stays with critical care lasting ≥ 1 day from January to July in 2020 vs. 2019

A total of 4874 paediatric admissions with at least one stay in a critical care unit were recorded in 2020 (1606 in P1, 958 in P2, and 2310 in P3), and 5302 were recorded in 2019 (1632 in P1, 1430 in P2, and 2240 in P3) (Table 2).

**Table 2:** Characteristics of paediatric patients admitted to a critical care unit for at least 1 day before, during, and after the first period of COVID-19 lockdown in France

	Before lockdown (P1)			During lockdown (P2)			After lockdown (P3)		
Characteristics	2019	2020	*p* value	2019	2020	*p* value	2019	2020	*p* value
All (*n*)	1632	1606		1430	958		2240	2310	
Age (year)			0.6621			< 0.0001			0.0057
Median (IQR)	10.0 (1.0–15.0)	10.0 (2.0–15.0)		10.0 (2.0–15.0)	5.0 (1.0–13.0)		10.0 (2.0–15.0)	8.0 (2.0–14.0)	
Range	0.0–17.0	0.0–17.0		0.0–17.0	0.0–17.0		0.0–17.0	0.0–17.0	
Age categories *n* (%)			0.2583			< 0.0001			0.0008
Below 1 year	421 (25.8)	386 (24.0)		296 (20.7)	298 (31.1)		467 (20.8)	515 (22.3)	
2–5 years	218 (13.4)	241 (15.0)		250 (17.5)	206 (21.5)		377 (16.8)	441 (19.1)	
6–12 years	314 (19.2)	285 (17.7)		307 (21.5)	183 (19.1)		475 (21.2)	539 (23.3)	
13–17 years	679 (41.6)	694 (43.2)		577 (40.3)	271 (28.3)		921 (41.1)	815 (35.3)	
Sex *n* (%)			0.2877			0.0693			0.1733
Female	760 (46.6)	718 (44.7)		603 (42.2)	440 (45.9)		918 (41.0)	901 (39.0)	
Length of hospital stay *n* (%)			0.4506			0.3496			0.0380
Median (IQR)	3.0 (1.0–8.0)	3.0 (1.0–9.0)		3.0 (1.0–8.0)	3.0 (1.0–9.0)		3.0 (1.0–7.0)	3.0 (1.0–7.0)	
Length of hospital stay (days, in classes) *n*, (%)			0.8608			0.0930			0.0559
1– < 2	471 (28.9)	460 (28.6)		389 (27.2)	298 (31.1)		731 (32.6)	722 (31.3)	
2– < 3	231 (14.2)	218 (13.6)		217 (15.2)	147 (15.3)		377 (16.8)	344 (14.9)	
≥ 3	930 (57.0)	928 (57.8)		824 (57.6)	513 (53.5)		1 132 (50.5)	1 244 (53.9)	
Death in hospital *n* (%)	25 (1.5)	33 (2.1)	0.2620	15 (1.0)	19 (2.0)	0.0589	36 (1.6)	30 (1.3)	0.3843
Admissions to a PICU *n* (%)	585 (35.8)	543 (33.8)	0.2243	479 (33.5)	332 (34.7)	0.5578	764 (34.1)	818 (35.4)	0.3558
Admissions to a paediatric intermediate care unit *n* (%)	1195 (73.2)	1185 (73.8)	0.7168	1053 (73.6)	710 (74.1)	0.7952	1629 (72.7)	1696 (73.4)	0.5963
Surgery *n* (%)	499 (30.6)	518 (32.3)	0.3037	424 (29.7)	295 (30.8)	0.5507	683 (30.5)	758 (32.8)	0.0922

*SD*, standard deviation; *IQR*, interquartile range; *PICU* paediatric intensive care unit

**Fig. 3:**
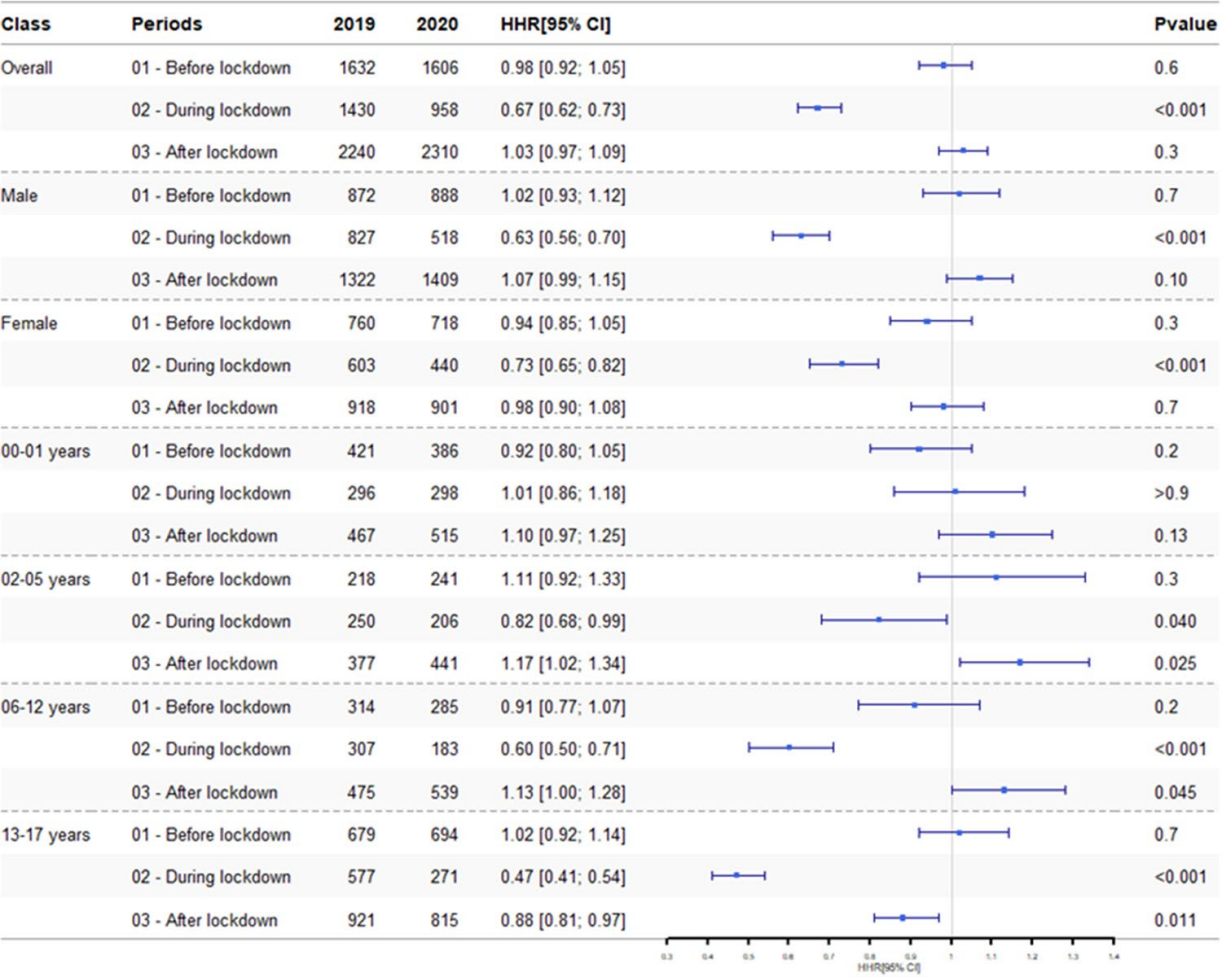
Hospital admission rate ratios (HRR) for paediatric accidents requiring critical care by age and by sex, from January 1 to July 31, 2019, and from January 1 to July 31, 2020

For P2, the risk of admission in critical care lasting for at least 1 day was lower in 2020 than in 2019 (HRR 0.67; 95% CI, 0.62–0.73; *p* < 0.001) (Fig. 3).

The median (IQR) length of stay was 3.0 (1.0–9.0) days in 2020 and 3.0 (1.0–8.0) days in 2019; this difference was not significant (*p* = 0.35). Likewise, the year-on-year difference in the proportion of patients having undergone surgery during P2 was not significant (30.8% in 2020 vs. 29.7% in 2019; *p* = 0.55).

For P1 and P3, the numbers of paediatric admissions with at least one stay in a critical care unit were similar in 2020 and 2019 (Fig. 3).

### Stratified analyses

The mean (SD) patient age was 8.7 (5.8) years in 2020 and 9.2 (5.7) years in 2019. When the absolute number of accidents was stratified by age, the lockdown period comprised fewer admissions in 2020 than in 2019 for all age groups (Tables 1 and 2, and Fig. 2). The largest decreases were observed for teenagers (aged 13–17; HRR, 0.34; 95% CI, 0.33–0.36; *p* < 0.001) and for 6- to 12-year-olds (HRR, 0.57; 95% CI, 0.55–0.59; *p* < 0.001). During P1, the number of admissions was similar in 2020 and 2019 for all age groups other than the 2- to 5-year-olds (HRR, 0.93; 95% CI, 0.89–0.97; *p* = 0.002). During P3, there were more admissions in 2020 than in 2019 for the youngest children (below 1 year of age; HRR, 1.06; 95% CI, 1.02–1.10; *p* = 0.002) and fewer admissions for 6- to 12-year-olds (HRR, 0.93; 95% CI, 0.90–0.95; *p* < 0.0001) and teenagers (HRR, 0.89; 95% CI, 0.87–0.91; *p* < 0.0001).

We next stratified the data for accident-related hospital stays involving at least 1 day of critical care by age group (Fig. 3): the most significant decrease from 2020 to 2019 during P2 was again observed for teenagers (HRR, 0.47; 95% CI, 0.41–0.54; *p* < 0.001) and 6- to 12-year-olds (HRR, 0.60; 95% CI, 0.50–0.71; *p* < 0.001). During P1, the numbers of accidents in 2020 vs. 2019 were similar for all age groups. During P3, there were more admissions with critical care in 2020 than in 2019 for the 2- to 5-year-olds (HRR, 1.17; 95% CI, 1.02–1.34; *p* = 0.025) and for 6- to 12-year-olds (HRR, 1.13; 95% CI, 1.00–1.28; *p* = 0.045).

The accident rates by diagnosis remained stable for children under 5 years old between 2019 and 2020. For those aged 6 to 12 years, a decrease in admissions for traumatic neck injuries was observed (from 24.7 to 18.6%), alongside an increase in injuries to the hip and thigh (from 17.5 to 21.3%). Among teenagers, there was a reduction in admissions for traumatic neck injuries (from 1.9 to 13.0%) and head injuries (from 14.8 to 8.9%). However, an increase in admissions for teenagers was noted for injuries to the hip and thigh (from 22.9 to 29.7%) and injuries to the ankle and foot (from 16.8 to 21.0%) (eTable 3).

## Discussion

To the best of our knowledge, this large, nationwide study using a hospital discharge database is the first to have investigated the impact of France’s first nationwide COVID-19 lockdown period on hospital admissions of children for accidents. The absolute number of admissions for accidents was 44% lower in 2020 than in the same period in 2019. The lockdown period was associated with a decrease in the admissions across all age groups, and the number of accident-related paediatric hospital stays involving critical care for at least 1 day was lower in 2020 than in 2019.

There are discrepancies between the literature data from single-centre and multicentre studies on paediatric accidents during lockdown. In a study of 16 paediatric emergency departments (EDs) in Italy, the mean incidence of injuries was significantly higher in 2020 (17.9%) than in 2019 (5.6%) [10]. These findings are consistent with earlier single-centre studies performed in EDs in Italy and Turkey [3, 11]; despite the overall drop in ED visits between 2019 and 2020, the proportion of injury-related visits increased from 14 to 20.9% [12]. In a retrospective, single-centre study at a level 1 trauma centre in Austria, Payr et al. found that (i) the percentage of paediatric fractures did not decrease during the lockdown period, and (ii) the proportion of paediatric patients with mild traumatic brain injuries increased significantly [13]. In a single-centre study in France, Claudet et al. also reported an increase in the proportion of ED admissions for injuries [5]. However, all of these studies also observed a drop in the total number of visits to paediatric EDs. Other single-centre and multicentre studies have found a decrease in trauma-related ED visits of up to 60% [4, 14–16]. The present study, using nationwide data, is the first to have shown a decrease in accident-related hospital admissions of children during a lockdown period in France. We included all accident-related hospital admissions in France (i.e. direct ward admissions and admissions through the ED). The discrepancies in the results may also be due to interstudy differences in methodologies, inclusion criteria (all accidents or not), the degree of adherence to lockdown restrictions in each country, and the type of lockdown (e.g. whether outdoor activities were allowed) [3].

The greater likelihood of accident among children during a period of lockdown could be explained not only by the greater proportion of time spent at home but probably also by the negative psychological effects of home containment on both children and parents/caregivers. However, we observed a decrease in accident-related hospitalizations. All age groups were affected by the reduction in the absolute number of accidents during the lockdown period, while the proportion of accidents relative to total hospitalizations remained stable. Furthermore, our findings are similar to those reported by Ferro et al. and Keays et al. [3, 14]. The lockdown may have increased the level of child supervision and decreased the likelihood of traumatic accidents, particularly among young children [3, 10]. The decrease in the number of mild traumatic brain injuries in children aged 6 to 17, as observed by Keays et al. was unprecedented over the past 28 years [14].

As found in the present study, literature data show that accident-related hospital admission requiring critical care for at least 1 day was less frequent in 2020 than in 2019. In a study of 37 German PICUs with an average of 1444 admissions per centre, Bruns et al. observed that the number of admissions due to accidents/injuries fell from an average of 366 per centre to 346. Moreover, Bruns et al. found a reduction in the incidence of neurosurgeries for patients hospitalized in a PICU, likely due to a reduction in severe traumatic brain injuries [17]. The numbers of admissions traffic for accidents and school accidents decreased, whereas the numbers of household and leisure accidents increased [17]. In a prospective study conducted during the 8-week lockdown, Bolzinger et al. showed that the rate of domestic and trampoline accidents increased, while sports- and locomotion-related accidents decreased [18]. The lower incidence of sports injuries previously described among teenagers might be due to more time spent at home and restrictions on outdoor activities [17]. However, the present study did not include data on the circumstances of the injuries.

In a study of 27 children’s hospitals in the USA, Delaroche et al. reported a 45.7% decrease in paediatric ED visits and a 33.1% decrease in injury- and poisoning-related visits during the pandemic period, compared with the preceding 2 years [4]. Similar results were found in other studies [19, 20]. In contrast, the results of Zhang et al.’s study in Canada showed significant relative and absolute increases in the numbers of unintentional, recreational, and intentional drug exposures recorded in the ED during the pandemic, compared to the pre-pandemic years. The greatest increases were seen among teenagers and children below the age of 5 [21]. Chang et al.’s study results were consistent with US literature showing a significant rise in children’s exposure to cleaning products and disinfectants during the lockdown [22, 23]. However, it was unclear whether the increase in calls to the poison control centre reflected excessive concern and reporting by parents (who were at home more during the pandemic) or a true rise in exposure. Our study did not include data on calls to poison control centres in France. However, the decrease in drug use among teenagers might be explained by the limited access to drugs during the lockdown.

### Strengths and limitations

The present population-based study had several strengths. Firstly, our inclusion of data from a large number of patients increased the study’s statistical power. Secondly, the use of well-characterized ICD-10 diagnostic codes (as used in previous studies on this topic) allowed for proper categorization of the patients’ accidents [1].

The study also had some limitations. Firstly, we lacked data on the circumstances surrounding the accidents, such as whether they occurred indoors or outdoors. Secondly, the type of drugs involved in admissions for ingestion and poisoning was not documented. Thirdly, using an inpatient database has limitations because many paediatric injuries are not hospitalized and are discharged from the ED or outpatient settings. Lastly, the study’s retrospective design meant that the admission criteria applied at the various centres’ PICU could not be standardized. However, it should be noted that the French healthcare system did not collapse during the first wave of the pandemic, and no triage was performed for acute cases and emergencies.

## Conclusions

Based on data from a nationwide cohort, the results of the present study suggest that France’s COVID-19 lockdown period was associated with a decrease in the number of hospital admissions for paediatric accidents. Further research could usefully examine how parents create a safe home environment for their children and could serve as the basis for a parent education program—even outside periods of lockdown.

## Supplementary Information

Below is the link to the electronic supplementary material.

Supplementary file1 (DOCX 38 KB)

Supplementary file2 (DOCX 13 KB)

Supplementary file3 (DOCX 15 KB)

Supplementary file4 (DOCX 16 KB)

## Data Availability

The data that support the findings of this study and the data sets analyzed in the present study can be obtained upon a reasonable request to the corresponding author.

## Abbreviations

COVID-19: Coronavirus disease 2019
PMSI: Programme de médicalisation des systèmes d’information
ICD-10: Classification of diseases and delated health problems, tenth revision
PICU: Paediatric intensive care unit
SD: Standard deviation
HRR: Hospital admission rate ratio
ED: Emergency departments
SMR: Standardized morbidity ratio
