# Supplementary material for: Impact of the COVID-19 lockdown period on hospital admissions for paediatric accidents: a French nationwide study
Source: Eur J Pediatr. 2024 Dec 4;184(1):63. doi: 10.1007/s00431-024-05900-0 (PMC11618190; doi:10.1007/s00431-024-05900-0)

For each class and his period, we estimate a Poisson Model.

The model is written as follows :

$$\log\left( \boldsymbol{Y} \right)\boldsymbol{=aX+c}$$

Where :

- **Y** is the **outcome**. That is to say the number of children injuries.
- **X** is the **predictor.** It is a dummy variable with two modalities : 0 for the period in 2019 and 1 for the period in 2020. The reference modality is the period 2020.
- **a** is the estimated coefficient
- **c** is the intercept

After estimation, We compute the incidence rate ratio (IRR). It is equal to the exponent of the estimated coefficient **a** :

$$\boldsymbol{IRR=}\boldsymbol{e}^{\boldsymbol{a}}$$

In this article, the **IRR** is called the hospitalisation rate ratio (**HRR**).

For example, for the class « Overall » and the period « 01-Before lockdown », the data are :

| **Y** | **X** |
| --- | --- |
| 26 268 | 0 |
| 25 584 | 1 |

We use the glm() function of the library stats of R to estimate the model. We obtain the following result :


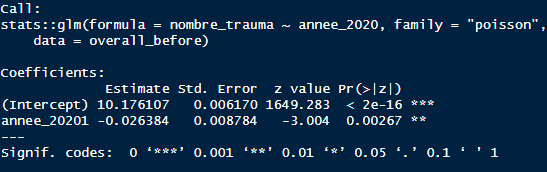


The variable **nombre_trauma** corresponds to the outcome **Y**.

The variable **annee_2020** corresponds to the predictor **X**.

After estimation, we compute the **HHR** and his **confidence interval** by using the tbl_regression() function of the library gtsummary of R. And, we obtain the following result :


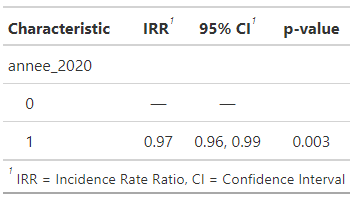

Supplement: Supplementary file 1 — Supplementary file1 (DOCX 38 KB) [file 431_2024_5900_MOESM1_ESM.docx]
