# Supplementary material for: Impact of the COVID-19 lockdown period on hospital admissions for paediatric accidents: a French nationwide study
Source: Eur J Pediatr. 2024 Dec 4;184(1):63. doi: 10.1007/s00431-024-05900-0 (PMC11618190; doi:10.1007/s00431-024-05900-0)
Supplement: Supplementary file 2 — Supplementary file2 (DOCX 13 KB) [file 431_2024_5900_MOESM2_ESM.docx]

| **Diagnosis** | **ICD-10 codes** |
| --- | --- |
| Traumatic neck injury | S10-S19 |
| Traumatic knee and leg injury | S80-S89 |
| Traumatic thoracic injury | S20-S29 |
| Traumatic injuries of the abdomen, lumbar spine and pelvis | S30-S39 |
| Traumatic injuries to several parts of the body | T00-T07 |
| Traumatic injuries of the hip and thigh | S70-S79 |
| Traumatic injuries of the elbow and forearm | S50-S59 |
| Traumatic injuries of the head | S00-S09 |
| Traumatic injuries of shoulder and arm | S40-S49 |
| Traumatic injuries to wrist and hand | S60-S69 |
| Traumatic injuries of unspecified site of trunk, limb or other body region | T08-T14 |
| Traumatic injuries of the ankle and foot | S90-S99 |
| Drowning and submersion | T751 |
| Effects of a foreign body entering a natural orifice | T15-T19 |
| Burns and corrosions | T20-T32 |
| Effects of electric current | T754 |
| Drug and biological poisoning | T36-T50 |
| Toxic effects of substances of essentially non-medicinal origin | T51-T65 |
