# Supplementary material for: Impact of the COVID-19 lockdown period on hospital admissions for paediatric accidents: a French nationwide study
Source: Eur J Pediatr. 2024 Dec 4;184(1):63. doi: 10.1007/s00431-024-05900-0 (PMC11618190; doi:10.1007/s00431-024-05900-0)
Supplement: Supplementary file 3 — Supplementary file3 (DOCX 15 KB) [file 431_2024_5900_MOESM3_ESM.docx]

| **Diagnosis** | **During lockdown (P2)** | |
| --- | --- | --- |
|  | **2019**  **n=23098**  **n (%)** | **2020**  **n=12864**  **n (%)** |
| S10-S19 Traumatic neck injury | 286 (1.2) | 87 (0.7) |
| S80-S89 traumatic knee and leg injury | 1977 (8.6) | 674 (5.2) |
| S20-S29 traumatic thoracic injury | 500 (2.2) | 201 (1.6) |
| S30-S39 Traumatic injuries of the abdomen, lumbar spine and pelvis | 973 (4.2) | 440 (3.4) |
| T00-T07 Traumatic injuries to several parts of the body | 80 (0.3) | 38 (0.3) |
| S70-S79 Traumatic injuries of the hip and thigh | 722 (3.1) | 370 (2.9) |
| S50-S59 Traumatic injuries of the elbow and forearm | 3690 (16.0) | 1950 (15.2) |
| S00-S09 Traumatic injuries of the head | 6908 (29.9) | 3789 (29.5) |
| S40-S49 Traumatic injuries of shoulder and arm | 1594 (6.9) | 979 (7.6) |
| S60-S69 Traumatic injuries to wrist and hand | 4112 (17.8) | 2531 (19.7) |
| T08-T14 Traumatic injuries of unspecified site of trunk, limb or other body region | 53 (0.2) | 35 (0.3) |
| S90-S99 Traumatic injuries of the ankle and foot | 537 (2.3) | 379 (2.9) |
| T751 Drowning and submersion | 47 (0.2) | 26 (0.2) |
| T15-T19 Effects of a foreign body entering a natural orifice | 686 (3.0) | 511 (4.0) |
| T20-T32 Burns and corrosions | 536 (2.3) | 532 (4.1) |
| T754 Effects of electric current | 52 (0.2) | 58 (0.5) |
| T36-T50 Drug and biological poisoning | 2041 (8.8) | 1023 (8.0) |
| T51-T65 Toxic effects of substances of essentially non-medicinal origin | 406 (1.8) | 282 (2.2) |
