# Supplementary material for: Impact of the COVID-19 lockdown period on hospital admissions for paediatric accidents: a French nationwide study
Source: Eur J Pediatr. 2024 Dec 4;184(1):63. doi: 10.1007/s00431-024-05900-0 (PMC11618190; doi:10.1007/s00431-024-05900-0)
Supplement: Supplementary file 4 — Supplementary file4 (DOCX 16 KB) [file 431_2024_5900_MOESM4_ESM.docx]

| **Diagnosis during lockdown period (P2)** | **00-01 year n (%)** | | **02-05 years n (%)** | | **06-12 years n (%)** | | **13-17 years n (%)** | |
| --- | --- | --- | --- | --- | --- | --- | --- | --- |
|  | **2019** | **2020** | **2019** | **2020** | **2019** | **2020** | **2019** | **2020** |
|  | **n=3151** | **n=2532** | **n=4152** | **n=3351** | **n=6895** | **n=3941** | **n=8900** | **n=3040** |
| S10-S19 Traumatic neck injury | 2055 (65.2%) | 1511 (59.7%) | 1554 (37.4%) | 1152 (34.4%) | 1704 (24.7%) | 732 (18.6%) | 1595 (17.9%) | 394 (13.0%) |
| S80-S89 traumatic knee and leg injury | 11 (0.3%) | 8 (0.3%) | 25 (0.6%) | 20 (0.6%) | 102 (1.5%) | 34 (0.9%) | 148 (1.7%) | 25 (0.8%) |
| S20-S29 traumatic thoracic injury | 34 (1.1%) | 41 (1.6%) | 50 (1.2%) | 34 (1.0%) | 136 (2.0%) | 52 (1.3%) | 280 (3.1%) | 74 (2.4%) |
| S30-S39 Traumatic injuries of the abdomen. lumbar spine and pelvis | 45 (1.4%) | 28 (1.1%) | 119 (2.9%) | 99 (3.0%) | 337 (4.9%) | 171 (4.3%) | 472 (5.3%) | 142 (4.7%) |
| T00-T07 Traumatic injuries to several parts of the body | 46 (1.5%) | 57 (2.3%) | 351 (8.5%) | 344 (10.3%) | 656 (9.5%) | 431 (10.9%) | 541 (6.1%) | 147 (4.8%) |
| S70-S79 Traumatic injuries of the hip and thigh | 215 (6.8%) | 233 (9.2%) | 657 (15.8%) | 557 (16.6%) | 1204 (17.5%) | 838 (21.3%) | 2036 (22.9%) | 903 (29.7%) |
| S50-S59 Traumatic injuries of the elbow and forearm | 86 (2.7%) | 63 (2.5%) | 140 (3.4%) | 114 (3.4%) | 197 (2.9%) | 101 (2.6%) | 299 (3.4%) | 92 (3.0%) |
| S00-S09 Traumatic injuries of the head | 53 (1.7%) | 55 (2.2%) | 124 (3.0%) | 88 (2.6%) | 479 (6.9%) | 261 (6.6%) | 1321 (14.8%) | 270 (8.9%) |
| S40-S49 Traumatic injuries of shoulder and arm | 7 (0.2%) | 7 (0.3%) | 11 (0.3%) | 10 (0.3%) | 17 (0.2%) | 10 (0.3%) | 45 (0.5%) | 11 (0.4%) |
| S60-S69 Traumatic injuries to wrist and hand | 7 (0.2%) | 11 (0.4%) | 12 (0.3%) | 6 (0.2%) | 16 (0.2%) | 11 (0.3%) | 18 (0.2%) | 7 (0.2%) |
| T08-T14 Traumatic injuries of unspecified site of trunk. limb or other body region | 167 (5.3%) | 123 (4.9%) | 302 (7.3%) | 248 (7.4%) | 163 (2.4%) | 107 (2.7%) | 54 (0.6%) | 33 (1.1%) |
| S90-S99 Traumatic injuries of the ankle and foot | 109 (3.5%) | 92 (3.6%) | 262 (6.3%) | 206 (6.1%) | 171 (2.5%) | 88 (2.2%) | 1499 (16.8%) | 637 (21.0%) |
| T751 Drowning and submersion | 30 (1.0%) | 16 (0.6%) | 65 (1.6%) | 79 (2.4%) | 149 (2.2%) | 159 (4.0%) | 293 (3.3%) | 125 (4.1%) |
| T15-T19 Effects of a foreign body entering a natural orifice | 80 (2.5%) | 60 (2.4%) | 112 (2.7%) | 91 (2.7%) | 96 (1.4%) | 78 (2.0%) | 118 (1.3%) | 53 (1.7%) |
| T20-T32 Burns and corrosions | 15 (0.5%) | 8 (0.3%) | 16 (0.4%) | 17 (0.5%) | 10 (0.1%) | 1 (0.0%) | 6 (0.1%) | 0 (0.0%) |
| T36-T50 Drug and biological poisoning | 42 (1.3%) | 38 (1.5%) | 398 (9.6%) | 383 (11.4%) | 1940 (28.1%) | 1086 (27.6%) | 1310 (14.7%) | 443 (14.6%) |
| T51-T65 Toxic effects of substances of essentially non-medicinal origin | 248 (7.9%) | 269 (10.6%) | 157 (3.8%) | 128 (3.8%) | 79 (1.1%) | 86 (2.2%) | 52 (0.6%) | 49 (1.6%) |
